# Deletion of fatty acid transport protein 2 (FATP2) in the mouse liver changes the metabolic landscape by increasing the expression of PPARα-regulated genes

**DOI:** 10.1074/jbc.RA120.012730

**Published:** 2020-03-18

**Authors:** Vincent M. Perez, Jeffrey Gabell, Mark Behrens, Nishikant Wase, Concetta C. DiRusso, Paul N. Black

**Affiliations:** ‡ Department of Biochemistry, University of Nebraska, Lincoln, Nebraska 68588; § Nebraska Center for Integrated Biomolecular Communication, University of Nebraska, Lincoln, Nebraska 68588

**Keywords:** liver, transcriptomics, lipid metabolism, metabolomics, gene regulation, fatty acid transport protein 2 (FATP2), metabolic landscape, peroxisome proliferator–activated receptor α (PPARα), RNA-Seq

## Abstract

Fatty acid transport protein 2 (FATP2) is highly expressed in the liver, small intestine, and kidney, where it functions in both the transport of exogenous long-chain fatty acids and the activation of very-long-chain fatty acids. Here, using a murine model, we investigated the phenotypic impacts of deleting FATP2, followed by a transcriptomic analysis using unbiased RNA-Seq to identify concomitant changes in the liver transcriptome. WT and FATP2-null (*Fatp2*^−/−^) mice (5 weeks) were maintained on a standard chow diet for 6 weeks. The *Fatp2*^−/−^ mice had reduced weight gain, lowered serum triglyceride, and increased serum cholesterol levels and attenuated dietary fatty acid absorption. Transcriptomic analysis of the liver revealed 258 differentially expressed genes in male *Fatp2*^−/−^ mice and a total of 91 in female *Fatp2*^−/−^ mice. These genes mapped to the following gene ontology categories: fatty acid degradation, peroxisome biogenesis, fatty acid synthesis, and retinol and arachidonic acid metabolism. Targeted RT-quantitative PCR verified the altered expression of selected genes. Of note, most of the genes with increased expression were known to be regulated by peroxisome proliferator–activated receptor α (PPARα), suggesting that FATP2 activity is linked to a PPARα-specific proximal ligand. Targeted metabolomic experiments in the *Fatp2*^−/−^ liver revealed increases of total C16:0, C16:1, and C18:1 fatty acids; increases in lipoxin A4 and prostaglandin J2; and a decrease in 20-hydroxyeicosatetraenoic acid. We conclude that the expression of FATP2 in the liver broadly affects the metabolic landscape through PPARα, indicating that FATP2 provides an important role in liver lipid metabolism through its transport or activation activities.

## Introduction

**Figure 1.**
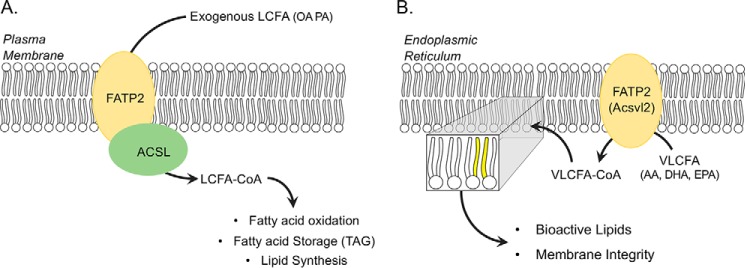
**Roles of FATP2 in fatty acid transport and very-long-chain fatty acid activation.** *A*, FATP2 functions at the plasma membrane in concert with a long-chain acyl-CoA synthetase (*ACSL*) to couple fatty acid transport and activation of long-chain fatty acids (*LCFA*; *e.g.* palmitate (*PA*) and oleate (*OA*)), resulting in LCFA-CoA, which enters downstream lipid metabolic pathways (*e.g.* fatty acid oxidation, fatty acid storage (triglycerides (*TAG*)), and lipid synthesis). *B*, FATP2 also functions as a very long chain acyl-CoA synthetase (Acsvl2) in the endoplasmic reticulum, where it activates VLCFAs (arachidonic acid (*AA*), docosahexaenoic acid (*DHA*), and eicosapentaenoic acid (*EPA*)). The resulting VLCFA-CoA enters downstream lipid metabolic pathways required for membrane integrity (shown as *yellow* acyl chains) and precursors for bioactive lipid synthesis.

Fatty acid transport protein 2/very-long-chain acyl-CoA synthetase 1 (FATP2;^2^ Slc27A2; Acsvl1) functions as a gatekeeper in its role as a fatty acid transport protein and a housekeeper in its role as a very-long-chain acyl-CoA synthetase required for metabolism of VLCFAs in anabolic and catabolic pathways. In cells and tissues where FATP2 is normally expressed (*i.e.* liver, intestine, and kidney), it appears to provide important roles in regulating the transport of exogenous fatty acids and in intracellular lipid homeostasis leading to structural and regulatory lipids and fatty acid mediators (Fig. 1) (1–5). The expression of FATP2 is controlled by both peroxisome proliferator–activated receptor α (PPARα) and FoxA1 (6, 7). Expression is increased in the liver under hypoxic conditions (8), in hepatocytes when Kupffer cells are depleted (9), and in hepatocytes under a high-fat diet (10).

Emerging evidence suggests that increased expression of FATP2 is linked to nonalcoholic fatty liver disease, renal disease, and some cancers (2, 11–13). Under conditions of lipid overload, increased expression of FATP2 in the liver leads to increased fat accumulation, inflammation, and organellar dysfunction (10, 14, 15). The selective inhibition of fatty acid (FA) transport using the FATP2-specific FA transport inhibitor, lipofermata, attenuates palmitate-induced lipotoxicity in HepG2 cells and depresses fatty acid absorption across the intestine in mice (2, 13, 16). In some cancers, the increased expression of FATP2 is correlated with the accumulation of triglyceride-rich lipid droplets and the promotion of metastasis, which may stem from increased FA transport (*e.g.* used for energy or membrane synthesis) or VLCFA activation for essential metabolic processes (*e.g.* membrane synthesis or synthesis of regulatory FA metabolites) (16–18). Polymorphonuclear myeloid-derived suppressor cells (PMN-MDSCs) are pathologically activated neutrophils and have increased expression of FATP2 that is associated with enhanced immunosuppressive activity (16, 19, 20). Deletion of FATP2 in PMN-MDSCs reduces tumor growth and is correlated with a reduction in the transport of arachidonic acid and the synthesis of prostaglandin E2 (16). The use of lipofermata slows tumor growth and proliferation, supporting the conclusion that FATP2 is specifically involved in regulating FA accumulation in PMN-MDSCs. In these different disease states, it is not fully understood how changes in FATP2 expression and/or activity (FA transport/VLCFA activation) specifically contribute to these pathologies.

Transporters and enzymes, including FATP2, do not act in isolation, but rather are essential in processes required to maintain metabolic homeostasis and, when dysfunctional through aberrant expression, contribute to different types of pathologies, including those noted above. Previous work from our laboratory defined the functional domains or motifs within the FATP family using directed mutagenesis of the yeast FATP orthologue, Fat1p (21). These studies showed that specific directed mutations within the two shared motifs, one required for ATP binding (ATP/AMP) and the other involved in fatty acid binding (FATP/VLACS), distinguished FA transport from VLCFA activation (21). When the mouse FATPs were expressed in a yeast strain defective in FA transport and activation, only the mouse FATP1, FATP2, and FATP4 functioned in FA uptake and VLCFA activation; FATP3, FATP5, and FATP6 did not function in FA uptake, further supporting the premise that functional elements within these proteins are distinguishable (22). Studies using mouse FATP1-FATP4 and FATP6-FATP4 protein chimeras expressed in yeast identified a 73-amino acid segment between the ATP/AMP and FATP/VLACS domains and common to FATP1 and FATP4 that contributes to FA transport (5, 23). The pivotal studies showing that the FA transport and VLCFA activation activities are distinct came from our studies addressing the role of FATP2 in trafficking exogenous fatty acids (3, 21). This study identified two splice variants of FATP2 (FATP2a/FATP2b) that functioned in FA transport and VLACS activation when expressed in yeast and 293T-REx cells; FATP2b lacks exon 2 that encodes the ATP/AMP binding domain and, while proficient in FA transport, was unable to activate VLCFAs (3, 21).

In the present work, we have used RNA-Seq to address the global impacts of deleting the *Fatp2* gene on the liver transcriptome to specifically understand how its expression influences the lipid metabolic landscape. Pathway enrichment analysis of the differentially expressed genes (DEGs) that were increased in the liver from FATP2-null mice (*Fatp2*^−/−^) showed enrichment in lipid metabolic pathways in the gene ontology categories of β-oxidation, peroxisome biogenesis, fatty acid biosynthesis, retinol metabolism, and arachidonic acid metabolism. These pathways are linked through PPARα, suggesting that FATP2 activity plays a unique role in the regulation of lipid metabolism in the liver, possibly through changes in the availability of a specific PPAR ligand. Natural ligands for PPARα include essential long-chain fatty acids (arachidonic and linoleic acid) and their metabolites.

## Results

### FATP2 is required for normal weight gain

**Figure 2.**
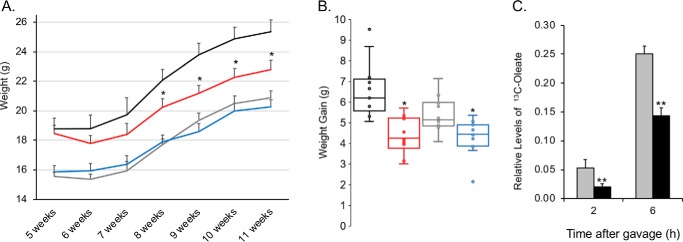
***Fatp2*^−/−^ mice gain less weight and have attenuated fatty acid absorption compared with the control mice (fed a standard chow diet).** *A*, weight gain of mice groups over the course of 6 weeks (beginning at 5 weeks of age until sacrifice at 11 weeks of age). Shown are control males (*black*) and females (*gray*) and *Fatp2*^−/−^ males (*red*) and females (*blue*); weight (g) ± S.D. (*error bars*) (*n* = 7); *, *p* ≤ 0.05 using Student's *t* test. *B*, quantification using box plots of weight gain over 6 weeks. Shown are control males (*black*) and females (*gray*) and *Fatp2*^−/−^ males (*red*) and females (*blue*); weight gain (g) ± S.D. (*n* = 7); *, *p* ≤ 0.05 using Student's *t* test. *C*, deletion of FATP2 attenuates fatty acid absorption. Male mice (control (*gray*) and *Fatp2*−/− (*black*)) were treated with 500 mg/kg [^13^C]oleate and then circulating blood levels of [^13^C]oleate measured after 2 and 6 h. Data are relative to ^12^C_18:1_ levels ± S.D. (*n* = 4); **, *p* ≤ 0.01 using Student's *t* test.

Both male and female *Fatp2*^−/−^ mice gained less weight compared with their control counterparts over the 6-week experimental period from 5 to 11 weeks of age (Fig. 2, *A* and *B*). This was most notable for the males, where there was a 25% smaller weight gain for the *Fatp2*^−/−^ mice when compared with controls. The weight gain by females was 10% less in the *Fatp2*^−/−^ mice (Fig. 2*B*). FATP2 is highly expressed in the small intestine, and our previous results showed that control mice treated with the FATP2-specific inhibitor, lipofermata, were compromised in their ability to absorb dietary fatty acids (13), and thus, we expected that *Fatp2*^−/−^ mice would result in comparable outcomes. Male mice were fasted, and following the intraperitoneal injection of tyloxapol to inhibit lipoprotein lipase-dependent systemic fatty acid uptake, they were gavaged with 500 mg/kg uniformly ^13^C-labeled oleate (C_18:1_) in flaxseed oil. The *Fatp2*^−/−^ mice, compared with control, had a 60% reduction in fatty acid absorption 2 h after lipofermata treatment and a 37% reduction 6 h after absorption (Fig. 2*C*). It is worth speculating that the decreased absorption of dietary fatty acids may be a contributing factor to the lower weight gain reported above.

Analysis of blood chemistry showed reductions in both circulating glucose and triglycerides in male and female *Fatp2*^−/−^ mice (Table 1). These changes were accompanied by significant increases in total cholesterol in both male and female mice. Whereas there were reductions in circulating glucose and triglycerides in both male and female mice, these reductions were only significant in the female *Fatp2*^−/−^ mice. It is unknown whether the reduction in triglycerides was directly linked to decreased fatty acid absorption from the intestines in the *Fatp2*^−/−^ mice. Alanine aminotransferase and aspartate aminotransferase levels were unchanged, suggesting no major impact on liver function due to FATP2 deletion.

**Table 1:** ***In vivo* circulating levels of glucose, lipids, and liver enzymes taken from retro-orbital bleeds** Values shown are mean ± S.D., *n* = 7 in each group. NS, not significant; *, *p* ≤ 0.05; **, *p* ≤ 0.01. ALT, alanine aminotransferase; AST, aspartate aminotransferase.

Serum parameter	Male control	Male *Fatp2*^−/−^	*p*	Female control	Female *Fatp2*^−/−^	*p*
Glucose (mg/dl)	254.7 ± 32.3	240.1 ± 28.3	NS	252.9 ± 19.1	226.3 ± 21.9	1.54E−02*
Triglycerides (mg/dl)	76.2 ± 4.9	72.6 ± 6.0	NS	60.2 ± 5.2	53.8 ± 5.7	3.45E−02*
Cholesterol (mg/dl)	94.0 ± 8.7	116.6 ± 6.7	1.35E−07**	85.1 ± 5.1	94.7 ± 4.2	1.54E−04**
ALT (units/liter)	104.6 ± 67.8	94.9 ± 36.7	NS	53.3 ± 15.1	50.6 ± 16.8	NS
AST (units/liter)	142.7 ± 33.8	127.9 ± 32.7	NS	93.7 ± 12.3	107.0 ± 31.9	NS

### Liver transcriptomes from control and Fatp2^−/−^ mice are distinguishable

**Figure 3.**
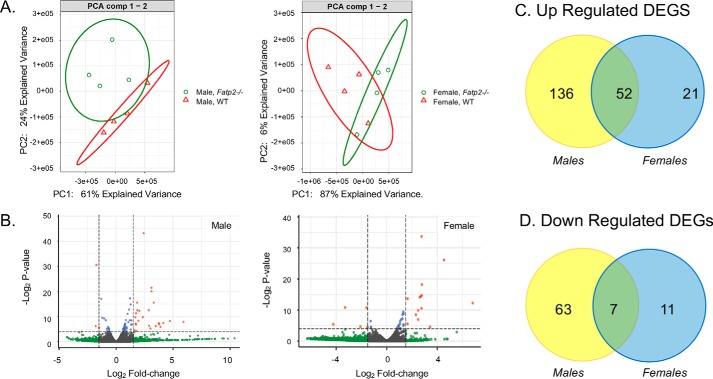
**WT and *Fatp2*^−/−^ liver transcriptomes are distinguishable.** *A*, PCA plots of gene counts from the liver transcriptomes from control (*red*) and *Fatp2*^−/−^ (*green*) male (*left*) and female (*right*) mice using unsupervised clustering (*n* = 4). *B*, volcano plots of genes from *Fatp2*^−/−^ male (*left*) and female (*right*) liver tissue generated using a log_2_ -fold change and corrected *p* values of the genes in the male and female RNA-Seq data. The genes in *red* meet both the *p* value cut-off (*p* ≤ 0.05) and log_2_ -fold change cut-off (≥1.5); genes in *blue* meet the *p* value cut-off but not log_2_ -fold change; and those in *gray* do not meet either the *p* value or the log_2_ -fold change threshold. (*n* = 4). *C*, up-regulated DEGs in the liver from male (*yellow*) and female (*blue*) *Fatp2*^−/−^ mice with 52 in common. *D*, down-regulated DEGs in the liver from male (*yellow*) and female (*blue*) *Fatp2*^−/−^ mice with seven in common.

**Figure 4.**
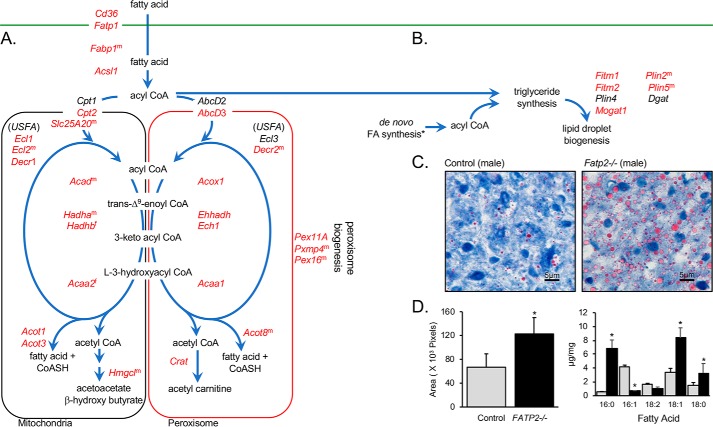
**DEGs in fatty acid transport, trafficking and oxidation, lipid droplet biogenesis, and peroxisome biogenesis correlate with increased size of lipid droplets in the *Fatp2*^−/−^ liver.** *A*, increased expression of genes (*red*) encoding proteins involved in fatty acid transport, trafficking, β-oxidation (mitochondrial (*left*) and peroxisomal (*right*)), and peroxisome biogenesis pathways. *B*, DEGs enriched in the pathways leading to triglyceride and lipid droplet biogenesis. For *A* and *B*, DEGs increased in expression are colored in *red*; for genes noted with *superscript m* or *f*, expression changed only in male or female mice, respectively; genes noted in *black* were detected but were unchanged between the control and *Fatp2*^−/−^ mice. *C*, control (*left*) and *Fatp2*^−/−^ (*right*) liver sections stained with Oil Red O; images are representative of *n* = 4 mice. *D*, quantification of Oil Red O staining (*left*, indicative of lipid droplets) as noted in Fig. 4*C* using ImageJ software; data are ± S.D. (*error bars*) (*n* = 4). Quantification of neutral lipids (*right*) as fatty acid methyl esters from control (*gray*) and *Fatp2*^−/−^ (*black*) liver (μg of FA/mg of tissue ± S.D. (*n* = 4); *, *p* ≤ 0.05 from Student's *t* test.

Whereas FATP2 is highly expressed in the liver and regulated by PPARα and FoxA1 (6, 24), its precise role in liver metabolism has not been fully established. As a first step in addressing the role of FATP2 in the liver, a nontargeted transcriptomic approach was employed; mRNA transcripts corresponding to 23,326 genes were identified and quantified (Tables S1–S3). The transcriptomes of both male and female mice of both genotypes (control and *Fatp2*^−/−^) were analyzed using an unsupervised principal component analysis (PCA) (Fig. 3*A*). The experimental groups were well-segregated and were differentially distinguished by both sex and genotype. Therefore, the male and female transcriptomes were further evaluated separately. We controlled for false discovery rates (FDR) using the Benjamini–Hochberg methods based on negative binomial distribution in DESeq (25). Volcano plots were generated showing -fold change (FC) and *p* values adjusted for FDR (Fig. 3*B*). Each plot illustrates DEGs that met the cut-offs for a *p* value (adjusted for FDR) of 0.05 and FC of 1.5. DEGs that were increased or decreased in expression and met the *p* value and FC cut-off (*p* ≤ 0.05 and |log_2_ FC| ≥ 1.5) are *colored red*, DEGs that only met the *p* value cut-off (*p* ≤ 0.05) are *colored blue*, DEGs that only met the FC cut-off (|log_2_ FC| ≥ 1.5) are *colored green*, and DEGs that met neither cut-off are *colored gray* (Fig. 4*B*). The male *Fatp2*^−/−^ mice had 258 total DEGs of which 188 were increased in expression and 70 decreased in expression relative to control values (Fig. 3*C* and Table S4). The impact of genotype was less pronounced in the female mice with a total of 91 DEGs, of which 73 were increased and 18 decreased in expression (Fig. 3*D*). There were only 59 DEGs shared between male and female *Fatp2*^−/−^ mice, and of these, 52 were increased and 7 decreased. These data may suggest some phenotypic differences in FATP2 activities that may be sex-dependent.

### KEGG enrichment analysis reveals increased expression of lipid metabolic genes in the Fatp2^−/−^ liver

The DEGs with increased expression clustered into several KEGG pathways related to fatty acid metabolism that included fatty acid degradation, PPAR signaling, peroxisome biogenesis, unsaturated fatty acid biosynthesis, retinol metabolism, and arachidonic acid metabolism (Table 2). Of the 258 DEGs with increased expression in male *Fatp2*^−/−^ mice, 107 clustered similarly into six KEGG enrichment pathways related to fatty acid metabolism: fatty acid degradation (18 genes), peroxisome biogenesis (20 genes), PPAR signaling (20 genes), retinol metabolism (11 genes), arachidonic acid metabolism (10 genes), and unsaturated fatty acid biosynthesis (8 genes) (Table 2). Of the 91 DEGs from the female *Fatp2*^−/−^ mice, 81 were likewise clustered into the same six KEGG enrichment pathways: fatty acid degradation (17 genes), PPAR signaling (15 genes), peroxisome biogenesis (13 genes), retinol metabolism (7 genes), arachidonic acid metabolism (5 genes), and unsaturated fatty acid biosynthesis (5 genes) (Table 2).

**Table 2:** **KEGG pathway enrichment of DEGs in male and female *Fatp2*^−/−^ mouse liver (*n* = 4)**

Term	Male and female DEGs	Male DEGs	Female DEGs	Total gene pathway
Fatty acid degradation	11	18	17	49
Peroxisome biogenesis	12	20	13	81
PPAR signaling	12	20	15	82
Biosynthesis of unsaturated fatty acids	5	8	5	25
Retinol metabolism	5	11	7	88
Arachidonic acid metabolism	3	10	5	90

To further examine the impact of deleting FATP2 in the liver, we next focused our analyses on specific pathways connected to fatty acid transport, trafficking, and oxidation (Fig. 4). Of interest was the finding that genes encoding fatty acid–binding protein 1 (*Fabp1*), CD36/fatty acid translocase (*Cd36*/*Fat*), *Fatp1*, and *Acsl1* each had 1.6- to nearly 6-fold increased expression in the *Fatp2*^−/−^ mice (Table 3 and Fig. 4*A*). CD36, FATP1, and Acsl1 function in the fatty acid transport pathway, as evidenced by studies showing that their heterologous overexpression increases fatty acid transport (26, 27). The deletion of FATP2 in the liver suggests there may be compensation at the level of fatty transport, as reflected by the nearly 4-fold and 6-fold increased expression of *Fatp1* and *Cd36*, respectively, and possibly *Fabp1*, which was increased 1.6-fold.

**Table 3:** **Selected DEGs in *Fatp2*^−/−^ mouse liver showing -fold change compared with WT** FC, -fold change; NS, not significant.

Gene	Description	Males		Females	
		FC	*p*	FC	*p*
**Fatty acid transport and trafficking**					
*Cd36*	CD36/FAT	5.9	7.18E−10	5.5	2.95E−23
*Fabp1*	Fatty acid–binding protein 1	1.6	7.11E−03		NS
*Fatp1*	Fatty acid transport protein 1	3.8	1.38E−22	3.1	2.22E−06
*Acsl1*	Acyl-CoA synthetase long-chain 1	2.0	4.62E−09	2.1	2.28E−04
**Fatty acid oxidation**					
Mitochondrial					
*Cpt2*	Carnitine palmitoyltransferase 2	1.5	2.52E−03	2.0	1.42E−03
*Slc25A20*	Solute carrier family 25A20	1.8	8.63E−06		NS
*AcadM*	Acyl-CoA dehydrogenase medium chain	NS		2.0	9.01E−04
*AcadL*	Acyl-CoA dehydrogenase long chain	NS		1.9	6.42E−03
*HadhA*	Hydroxyacyl-CoA dehydrogenase A	1.4	4.33E−02	NS	
*HadhB*	Hydroxyacyl-CoA dehydrogenase B		NS	1.8	4.46E−02
*Acaa2*	Acetyl-CoA acyltransferase 2		NS	1.8	1.78E−02
*Acot1*	Acyl-CoA thioesterase 1	8.6	9.68E−19	6.8	6.29E−30
*Acot3*	Acyl-CoA thioesterase 3	8.1	1.18E−03	2.1	4.16E−02
*Hmgcl*	3-Hydroxy-3-methylglutaryl-CoA lyase	1.5	1.30E−02		NS
*Ecl1*	Enoyl-CoA Δ-isomerase 1	1.8	2.26E−09	1.9	5.37E−06
*Ecl2*	Enoyl-CoA Δ-isomerase 2	2.0	7.05E−12		NS
*Decr1*	2,4-Dienoyl CoA reductase 1	1.7	6.07E−05	2.0	1.61E−03
*Etfdh*	Electron-transferring flavoprotein	NS		1.9	5.34E−03
Peroxisomal					
*AbcD3*	ATP-binding cassette D3	1.8	2.10E−06	2.2	2.21E−05
*Acox1*	Acyl-CoA oxidase 1, palmitoyl	2.3	9.75E−15	2.5	1.69E−06
*Acaa1A*	Acetyl-CoA acyltransferase 1A	1.6	3.34E−04	1.8	4.39E−02
*Acaa1B*	Acetyl-CoA acyltransferase 1B	3.5	1.42E−14	4.4	5.74E−18
*Ecl1*	Enoyl-CoA Δ-isomerase 1	1.8	2.26E−09	1.9	5.37E−06
*Ecl2*	Enoyl-CoA Δ-isomerase 2	2.0	7.05E−12		NS
*Ehhadh*	Enoyl-CoA, hydratase/dehydrogenase	5.4	3.57E−40	6.1	1.75E−11
*Acot8*	Acyl-CoA thioesterase 8	1.8	1.14E−04		NS
*Crat*	Carnitine acetyltransferase	3.3	1.32E−26	2.7	3.94E−07
*Decr2*	2–4-Dienoyl-CoA reductase 2	1.6	6.82E-04		NS
**Fatty acid synthesis**					
*Me1*	Malic enzyme 1, NADP^+^-dependent	1.7	1.40E−02		NS
*Scd1*	Stearoyl-CoA desaturase 1	1.9	3.77E−02		NS
*Elovl5*	Fatty acyl-CoA elongase 5	1.4	3.69E−02		NS
**Lipid body biogenesis**					
*Fitm1*	Fat storage-inducing transmembrane protein1	3.4	4.54E−05	6.8	5.91E−12
*Fitm2*	Fat storage–inducing transmembrane protein2	1.9	1.76E−06		NS
*Plin2*	Perilipin 2	2.0	5.72E−09		NS
*Plin5*	Perilipin 5	1.9	1.19E−06		NS
*Mogat1*	Acyl-CoA:monoacylglycerol acyltransferase	26.4	2.30E−06	111.2	1.02E−09
**Arachidonic acid metabolism**					
*Pla2*	Phospholipase A2	1.7	4.90E−05		NS
*Cyp4A10*	Cytochrome P450, family 4a10	32.9	1.75E−52	11.6	2.52E−10
*Cyp4A14*	Cytochrome P450, family 4a14	407.3	2.47E−63	30.5	5.74E−18
*Cyp4A32*	Cytochrome P450, family 4a32	20.9	6.22E−08	5.9	4.45E−05
**Retinol metabolism**					
*Dhrs4*	Dehydrogenase/reductase member 4	1.6	5.13E−07	1.9	8.86E−06
*Aldh1A1*	Aldehyde dehydrogenase 1A1		NS	2.1	2.40E−03
*Retsat*	Retinol saturase, *trans*-retinol 13,14-reductase	3.7	3.22E−15	7.7	1.45E−12
**Peroxisome biogenesis**					
*Pex11A*	Peroxisomal biogenesis factor 11A	2.3	3.88E−08	2.1	1.63E−03
*Pex16*	Peroxisomal biogenesis factor 16	1.5	8.37E−03		NS
*PxmP4*	Peroxisomal membrane protein	1.6	5.36E−04		NS

Coincident with increased expression of genes involved in fatty acid transport (*Cd36 and Fatp1*), trafficking (*Fabp1*), and activation (*Acsl1*), expression of mitochondrial and peroxisomal β-oxidation genes were also increased in the *Fatp2*^−/−^ liver (Fig. 4*A*). This suggests that deletion of *Fatp2* results in induction of fatty acid oxidation pathways in liver. Among these, there were notable sex-specific differences in the expression of several genes in the β-oxidation pathway (*e.g. Hmgcl*, *Acot8*, *Acad*, and *HadhB*) and in peroxisome biogenesis and function (*Pxmp4* and *Pex16*), whereby the fatty acid oxidation genes were more highly expressed in males over females. This seems to correlate with the sex-specific differences in weight gain (Table 3 and Fig. 2).

In the context of peroxisomal β-oxidation, there was also increased expression of key genes involved in peroxisomal fatty acid oxidation and proliferation (*e.g. Pex11a*, *Pex16*, *Pxmp4*, *Acaa1a*, *Acaa1b*, *Acot8*, *Acox1*, *Decr2*, *Ech1*, *Ehhadh*, and *Hacl1*). Of particular interest were increases in the three acyl-CoA thioesterases (*Acot1*, *Acot3*, and *Acot8*), expressed in the mitochondria and peroxisome, respectively, suggesting that deleting FATP2 in the liver may increase intracellular free FA levels (28).

**Figure 5.**
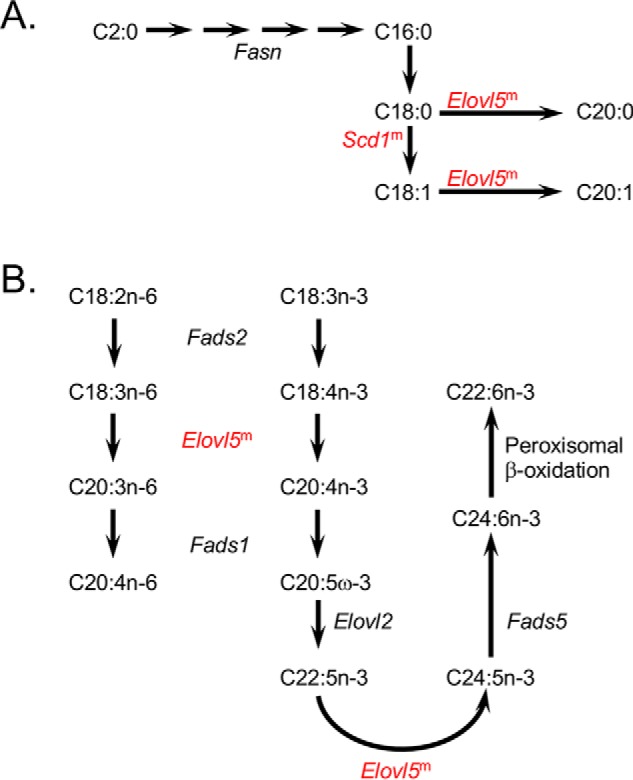
**Fatty acid synthesis pathways enriched in DEGs of the *Fatp2*^−/−^ liver.** *A*, DEGs in *de novo* fatty acid biosynthesis and fatty acid elongation. *B*, DEGs in essential fatty acid (C18:2 and C18:3) metabolism. For *A* and *B*, DEGs increased in expression are *colored* in *red*; for genes noted with *superscript m*, expression changed only in male mice; the genes noted in *black* were detected but were unchanged between the control and *Fatp2*^−/−^ mice.

Also noted from the RNA-Seq data were increases in several genes involved in lipid body formation in the *Fatp2*^−/−^ mouse livers (*e.g. Fitm1*, *Fitm2*, *Plin2*, *Plin5*, and *Mogat1*) (Fig. 4*B*). *Mogat*, encoding acyl-CoA:monoacylglycerol acyltransferase, was increased 26–111-fold (males and females, respectively), suggesting that deletion of *Fatp2* increased fatty acid flux from the diet into triglyceride synthesis. The genes encoding perilipins 2 and 5 (*Plin2* and *Plin5*) were increased about 2-fold. These genes encode the major lipid-droplet marker proteins that stabilize lipid droplets (29, 30). To address whether the increased expression of these genes corresponded to increased lipid body formation in the liver, we compared control and *Fatp2*^−/−^ sections of liver stained with Oil Red O (Fig. 4*C*). In line with these findings, there was a significant increase in lipid body staining with Oil Red O, which was quantified using ImageJ and correlated with increased total liver triglyceride levels enriched in palmitic acid (C16:0), stearic acid (C18:0), and oleic acid (C18:1) (Fig. 4*D*). Increases in these fatty acids correlate with increased expression of genes encoding enzymes of the unsaturated fatty acid biosynthesis and fatty acid elongation pathways in the *Fatp2*^−/−^ liver, including stearoyl-CoA desaturase-1 (*Scd1*, 2-fold) and fatty acid elongase 5 (*Elovl5*, 1.4-fold), although the increases in expression of these genes were only statistically significant in male *Fatp2*^−/−^ livers (Fig. 5). The increased expression of *Scd1* may contribute to the 2-fold increase in C18:1 in the liver triglyceride pools of the *Fatp2*^−/−^ mice reported above, as SCD1 is a Δ^9^ desaturase for 12–19 carbon acyl-CoAs in rodents (31).

### Deletion of FATP2 in the liver changes the expression of genes involved in arachidonic acid and retinol metabolic genes and cancer progression

**Figure 6.**
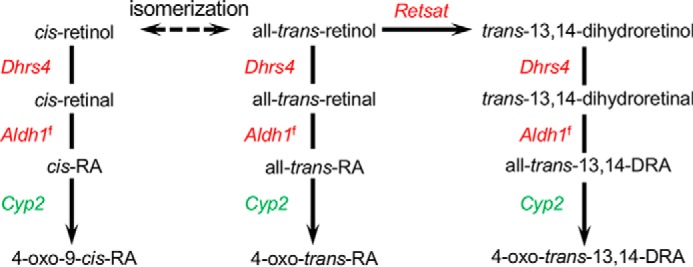
**Retinol metabolic pathway is enriched in DEGs of the *Fatp2*^−/−^ liver.** DEGs increased or decreased in expression are *colored* in *red* and *green*, respectively; for genes noted with *superscript f*, expression changed only in female mice.

**Figure 7.**
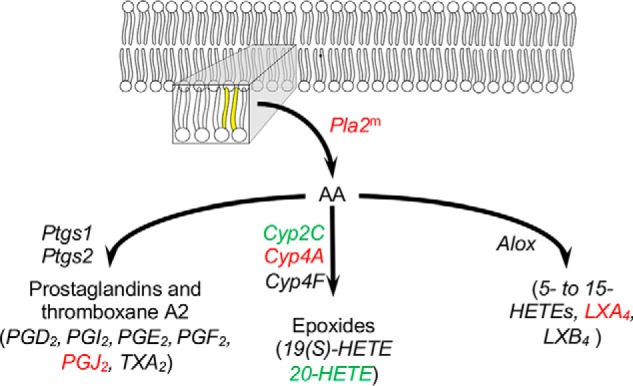
**Arachidonic acid metabolic pathway is enriched in DEGs of the *Fatp2*^−/−^ liver.** DEGs increased or decreased in expression are *colored* in *red* and *green*, respectively; for genes noted with *superscript m*, expression changed only in male mice; the genes noted in *black* were detected but were unchanged between the control and *Fatp2*^−/−^ mice. The acyl chains *colored* in *yellow* indicate arachidonic acid (*AA*) in the membrane that is released by Pla2. Metabolites measured are *italicized*, showing those with increased (*red*), decreased (*green*), or unchanged (*black*) levels.

Transcriptome analysis identified changes in genes encoding enzymes involved in both arachidonic acid and retinol metabolism in male and female *Fatp2*^−/−^ mouse livers (Table 3 and Figs. 6 and 7). For retinol metabolism, the oxidoreductase retinol saturase gene (*Retsat*), which has been shown to correlate with the development of fatty liver disease, had a ∼4-fold increase in the male livers and an ∼8-fold increase in the female livers (Fig. 6) (32). In the case of arachidonic acid metabolism, several cytochrome p450 family 4 (*Cyp4A*) genes involved in bioactive lipid synthesis had increased expression, including *Cyp4A32*, *Cyp4A10*, and *Cyp4A14*, which, in male mice, was coincident with an approximately 2-fold increase in the expression of phospholipase A2 (*Pla2*) (Fig. 7). In contrast to the increased expression of several *Cyp4a* genes, it was of interest to find that there was decreased expression of genes encoding the cytochrome p450 family 2 (CYP2) enzymes in the male *Fatp2*^−/−^ liver, but not the female liver (Table S4).

Several additional genes, which were decreased in expression in the *Fatp2*^−/−^ liver, were notable. Whereas these did not map to specific KEGG pathways, several have been implicated in cancer progression (Table S4). Among these, the expression of TNFα receptor–associated factor (TRAF)-interacting protein with a forkhead-associated (FHA) domain (TIFA) was significantly decreased in the *fatp2*^−/−^ livers. High expression of this protein is associated with poor prognosis in lung adenocarcinoma (33). The expression of TIFA is also increased in acute myeloid leukemia (34). Likewise, there was increased expression of interleukin 28 receptor, α (IL22RA1), which is associated with stage II colon cancer (35). These findings suggest that in these cancers, increases in FATP2 activity may contribute to cancer establishment and progression. This is consistent with recent studies reporting that *Fatp2* is highly expressed in PMN-MDSCs of several cancer model systems. Tumor size and cell proliferation was reduced in *Fatp2*^−/−^ mice and in control mice treated with the FATP2-specific inhibitor lipofermata (16, 19).

### RT-qPCR analyses confirm differential gene expression levels defined using RNA-Seq in Fatp2^−/−^ mouse livers

To verify the changes in gene expression in the liver from *Fatp2*^−/−^ mice using RNA-Seq, we employed RT-qPCR of genes selected on the basis of their roles in fatty acid synthesis, transport, activation, and trafficking (Fig. S1). Normalized RNA expression relative to *Gapdh* was used to calculate log_2_ FC of 15 genes in the *Fatp2*^−/−^ mice *versus* controls using RNA from livers of mice of both genotypes and sexes (30 measurements total). The resultant values were plotted against the log_2_ FC values of the corresponding genes from RNA-Seq data (Fig. S1). The log_2_ FC from both experiments correlated well, with an *R*^2^ of 0.74 using a best-fit linear trendline. Among the genes evaluated, *Fabp1, Cyp4A10*, and *Cd36* were confirmed to be increased in expression in both male and female liver. *Fabp1* was increased 1.7-fold in males and 2.4-fold in females. *Cyp4a10* was increased 13-fold in males and 15-fold in females. *Cd36* expression was increased 5-fold in both males and females. The data confirm the findings from global RNA-Seq experiments as reported herein.

### Deletion of FATP2 changes PPARα signaling networks in the liver

**Figure 8.**
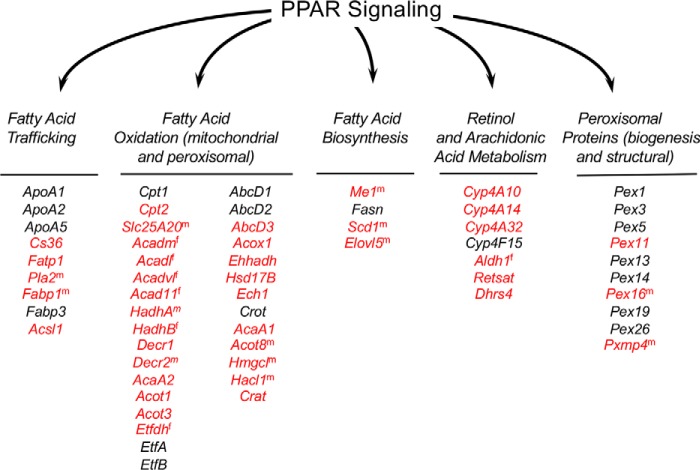
**Genes regulated through PPARα are increased in *Fatp2*^−/−^ mice.** All PPARα-regulated genes detected in the transcriptomic data set are noted and clustered into pathways related to fatty acid trafficking, fatty acid oxidation, fatty acid biosynthesis, retinol and arachidonic acid metabolism, and peroxisome proteins. Genes noted in *red* have increased expression in the *Fatp2*^−/−^ mice; those in *black* were detected but were unchanged when compared with the isogenic controls. *Superscript m* or *f* indicates that the expression of the noted gene was increased only in male or female liver, respectively.

Analysis of the RNA-Seq data showed that many DEGs mapped to pathways regulated by PPARα signaling for both *Fatp2*^−/−^ male and female mice. This suggests that the activities of FATP2 influence the transcriptional activity of PPARα. Of particular note was the increased expression of genes involved in fatty acid trafficking, fatty acid oxidation, fatty acid biosynthesis, retinol and arachidonic acid metabolism, and peroxisomal biogenesis (Table 3 and Fig. 8) (24). Given these findings, we addressed whether the deletion of FATP2 changed PPARα expression. Whereas there were slight increases in *Ppar*α expression (1.1 FC males, 1.3 FC females) in the *Fatp2*^−/−^ mice, after adjusting *p* values for FD, they lacked significance. Whereas these data only inform on changes in gene expression and not protein abundance or activity, they do suggest that the expression of FATP2 in the liver directly contributes to regulating the lipid metabolic landscape, likely through PPARα.

### Deletion of FATP2 alters hepatic fatty acid and eicosanoid levels

**Figure 9.**
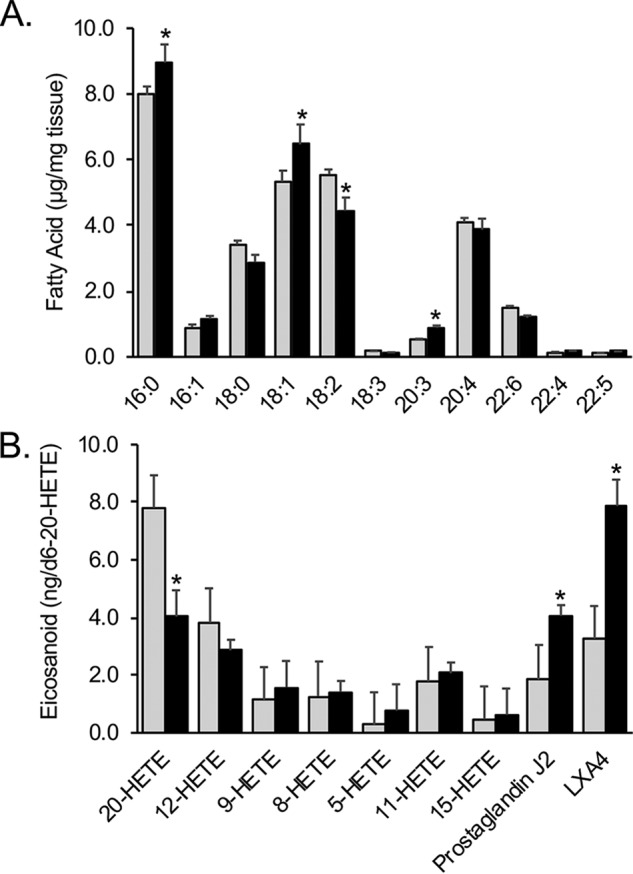
**The *Fatp2*^−/−^ mouse liver has altered total fatty acid and eicosanoid levels.** *A*, total fatty acids (mean ± S.D. (*error bars*), μg/mg) in male control (*gray*) and *Fatp2*^−/−^ (*black*) liver; *n* = 4; *, *p* ≤ 0.05 using Student's *t* test. *B*, total eicosanoid levels in male control (*gray*) and *Fatp2*^−/−^ (*black*) livers relative to the internal standard, *d*_6_-20-HETE ± S.D. (*n* = 5); *, *p* ≤ 0.05 from Student's *t* test.

The RNA-Seq data suggested that the changes in gene expression in the liver of *Fatp2*^−/−^ mice would likely contribute to changes in lipid metabolism. As noted above, the increased expression of *Plin1* and *Plin5* were correlated with increased lipid droplet formation in the liver, which was coincident with an increase in total triglyceride content. Deleting FATP2 in the liver resulted in a 20% increase in total C16:0 and C18:1 abundance (Fig. 9*A*), which was consistent with changes in the triglyceride pool, except for 18:0 (*e.g.* see Fig. 4*D*).

As noted above, the RNA-Seq data showed that key *Cyp4a* genes had increased expression (between 20- and 407-fold without log transformation) in the *Fatp2*^−/−^ mouse liver. These genes encode enzymes involved in eicosanoid biosynthesis using VLCFAs (*e.g.* arachidonic acid (C20:4) and docosahexaenoic acid (C22:6)) generated from phospholipids (likely phosphatidylcholine) through *Pla2*, which is overexpressed in the male *Fatp2*^−/−^ mouse liver (Fig. 7). Targeted metabolomic experiments using LC-MS/MS identified and quantified nine eicosanoids in control and *Fatp2*^−/−^ liver (Fig. 9*B*). Whereas levels of most were generally unchanged, there were statistically significant increases in prostaglandin J2 (PGJ2) and lipoxin A4 (LXA4) levels. There was a similar decrease in 20-HETE levels in the *Fatp2*^−/−^ livers.

## Discussion

This work demonstrates that FATP2 (*Slc27a2*, *Acsvl1*) plays a pivotal role in hepatic lipid metabolism and through its activities (FA transport and/or VLCFA activation) is linked to PPARα in regulating the expression of a number lipid metabolic genes. FATP2 was originally defined as a very-long-chain acyl-CoA synthetase (*Acsvl1*) with a limited role in the pathophysiology of X-linked adrenoleukodystrophy (36–38). Heinzer *et al.* (36) generated the FATP2/ACSVL1 knockout mouse (*Fatp2*^−/−^) and demonstrated elimination of very-long-chain acyl-CoA synthetase activity in the liver. The two activities of FATP2, long-chain FA transport and very-long-chain FA activation, are separable, as demonstrated in previous work from this laboratory using protein chimeras as well as two splice variants of FATP2 (FATP2a and FATP2b) (3, 22, 23). The locations of the functional domains were assigned and confirmed using site-directed mutagenesis of the Fat1p (FATP yeast orthologue) (3). In addition, decreased expression of FATP2 in liver using shRNA reduces fatty acid uptake (39). The present work extends these mechanistic findings and shows that the *Fatp2*^−/−^ mouse has reduced weight gain, attenuated uptake of exogenous fatty acids across the intestine, and increased expression of several key lipid metabolic genes in the liver. Importantly, the results reported here show that the expression of FATP2 is directly tied to PPARα activity.

The rationale for the current study using RNA-Seq to evaluate the liver transcriptome using control and *Fatp2*^−/−^ mice was to more fully understand the two functional activities of FATP2 and provide insights into how this protein functions in normal hepatic lipid metabolism and, when altered, contributes to metabolic dyshomeostasis leading to disease. Of particular note, these studies identified 42 lipid metabolic genes regulated by PPARα with expression significantly increased in the *Fatp2*^−/−^ mouse liver consistent with the notion that the functional activities of FATP2 impact the lipid metabolic regulatory landscape.

The RNA-Seq data identified key genes with increased expression in the fatty acid trafficking, fatty acid oxidation (mitochondrial and peroxisomal), and lipid body formation pathways in the *Fatp2*^−/−^ mouse liver. These metabolic pathways are linked through acyl-CoA, which can move into mitochondrial and peroxisomal β-oxidation and into lipid biosynthetic pathways, including triglyceride synthesis. It was of interest to find that the genes encoding fatty acid transporters CD36 and FATP1 and the long-chain acyl-CoA synthetase, Acsl1, were increased in expression in the *Fatp2*^−/−^ liver transcriptome. Previous work from a number of laboratories has shown that when CD36, FATP1, or Acsl1 is overexpressed, there is an increase in fatty acid transport (26, 27, 40). Given the increased expression of these genes in the *Fatp2*^−/−^ mouse liver, it is reasonable to consider that there may be at least partial compensation for the loss of FATP2-mediated FA transport. This is suggested to be the case, because lipid accumulation and lipid body formation were noted in *Fatp2*^−/−^ livers. Further, RNA-Seq data showed that in the *Fatp2*^−/−^ mouse liver, there are increases in expression of two key perilipins, perilipin 2 and 5, which was coincident with the increase in lipid body accumulation. PLIN2 is a major lipid droplet–associated protein (30), whereas PLIN5 is selectively expressed in tissues where fatty acids are released during lipolysis and transported to mitochondria for β-oxidation (29, 41). Under fasting conditions, lipid bodies accumulate in the liver (42, 43), and thus deletion of FATP2 may emulate such conditions.

Under fasting conditions, liver fatty acid oxidation and ketone body synthesis increase to provide energy and to spare glucose oxidation in peripheral tissues. The *Fatp2*^−/−^ livers had increased expression of genes encoding β-oxidation enzymes along with 3-hydroxy-3-methylglutaryl-CoA lyase (HMG-CoA synthase, *Hmgcl* gene), the rate-limiting enzyme of ketone body biosynthesis (44). HMG-CoA synthase is regulatory in the ketogenic process and increased under fasting conditions (45). We speculate that these activities contribute to the decrease in weight gain and perhaps the reduced plasma triglycerides, also mimicking the fasting condition, in the *Fatp2*^−/−^ mice.

Analysis of the RNA-Seq data also identified three additional networks with increased expression of key metabolic genes in the *Fatp2*^−/−^ mouse liver controlled through PPARα. The first was unsaturated fatty acid biosynthesis indicated by increased expression of stearoyl CoA desaturase (*Scd1*) and fatty acid elongase (*Elovl5*) (24, 46). Whereas fatty acid synthase (*Fasn*) was identified in the gene sets, its expression remained essentially unchanged in the *Fatp2*^−/−^ mouse liver. *Fasn* gene expression is activated in a ChREBP- and SREBP1c-dependent manner (47). The increased expression of *Scd1* may contribute to increasing C18:1 level, especially in the liver triglycerides, reflected by increases in lipid body formation. The second notable pathway was retinol metabolism. Deletion of *Fatp2* resulted in increased expression of the dehydrogenase/reductase 4 (*Dhrs4*), *Retsat*, and aldehyde dehydrogenase 1 (*Aldh1*) genes. Increased expression of these enzymes is expected to result in increased levels of 9-*cis*-retinoic acid, a ligand for RXR, a binding partner of PPARα (48), thus driving increased expression of PPARα-regulated genes (49). The third PPARα-responsive pathway is arachidonic acid metabolism. In this case, genes encoding *Cyp4* family enzymes were increased in expression when *Fatp2* was deleted. Increased activities of these enzymes, coincident with increased expression and activity of phospholipase A2 (*Pla2*), would be expected to increase free arachidonic acid for use as a substrate in eicosanoid synthetic pathways. This would explain the measured increases of PGJ2 and LXA4 (50–52).

When FATP2 expression is increased in the presence of exogenously added palmitate (C16:0), it becomes localized to the plasma membrane and is correlated with the development of lipotoxicity (10). In addition, increased expression of FATP2 is associated with the progression of nonalcoholic fatty liver disease (11). The PPARα agonists Wy12643 and GW7647 alter *Fatp2* expression, whereas in the PPARα-null mice, its expression is increased (7, 53). The increased expression of the pioneer factor FOXA1 in HepG2 cells and primary hepatocytes decreases *Fatp2* expression along with lowered levels of fatty acid transport, thus protecting the liver from steatosis (6). Adenovirus-directed *Fatp2* shRNA reduces fatty acid uptake and protects mice from diet-induced nonalcoholic fatty liver disease (39). The FATP2-specific inhibitor, lipofermata, attenuates fatty acid uptake across the intestine in mice and blocks the development of lipotoxicity in HepG2 cells (2, 4). Lipofermata functions as a noncompetitive inhibitor that specifically attenuates the uptake of long-chain fatty acids and prevents cellular dysfunction and death caused by saturated fatty acids (2).

More recently, Veglia *et al.* (16) demonstrated that PMN-MDSCs have increased expression of *Fatp2*, which is correlated with immunosuppressive activity in cancer. Deletion of *Fatp2* in PMN-MDSCs significantly reduces tumor growth when compared with isogenic controls. Further, the FATP2-specific FA transport inhibitor lipofermata attenuates the activity of PMN-MDSCs and delays of tumor progression (16). Several genes involved in cancer progression in different tissue types were decreased in the *Fatp2*^−/−^ liver, suggesting a linkage between cancer progression and FATP2 expression (19). There is evidence that in PMN-MDSCs FATP2 functions in the transport of arachidonic acid, which may be trafficked into downstream eicosanoid synthesis pathways. In the present work, we have shown that deletion of FATP2 alters the transcriptional landscape of genes that are largely regulated through PPARα. However, the relationships between *Fatp2* expression and changes on the eicosanoid synthesis pathways are not yet fully understood.

FATP2 appears to play an important role in both fatty acid transport and VLCFA activation and through one or both activities is linked to PPARα in regulating the lipid metabolic landscape in the liver. Whereas there are limitations to RNA-Seq and next generation sequencing by being restricted to transcriptional output, we have uncovered important information showing that the expression of *Fatp2* is tightly linked to the hepatic lipid metabolic landscape. Our current efforts are working to address the impact of a high-fat diet on the lipid metabolic landscape using the *Fatp2*^−/−^ mouse model. Additionally, we are focused on using lipofermata in combination with the *Fatp2*^−/−^ mouse to distinguish the function of FATP2 in FA transport from VLCFA activation in the context of these PPARα regulatory networks.

## Experimental procedures

### Animal care and diets

C57BL/6 male and female mice (6 weeks post-weaning) were obtained from Jackson Laboratories and allowed to acclimate 1 week prior to the experiments. C57BL/6 *Fatp2*^−/−^ mice were obtained by back-crossing SV129 *Fatp2*^−/−^ mice (36) into the C57BL/6 strain of mice over 10 generations. The *Fatp2* deletion encompasses 7 kb and contains exon 2, intron 2, a neomycin-resistance cassette in place exon 3, intron 3, and exon 4 (36). The neomycin-resistance cassette increases the size of a HinDIII genomic DNA fragment detected using a probe outside of exon 2 and routinely used to monitor the *Fatp2*^−/−^ mouse colony. This construct effectively eliminates the expression of both FATP2a and FATP2b. Genotypes were confirmed using PCR amplification and qPCR. Control and *Fatp2*^−/−^ male and female mice were aged-matched and housed (3 mice/cage) in an AAALAC-accredited research facility at the University of Nebraska (Lincoln, NE) in ventilated cages at 22 °C with a 14/10-h light/dark cycle. Mice were allowed *ad libitum* access to water and a standard rodent chow (Teklad, 2016 16% Protein Rodent Diet). Animals were sacrificed using CO_2_ narcosis followed by cervical dislocation. For RNA isolation, liver samples were placed in RNAlater® (Ambion, Inc.) and immediately snap-frozen using liquid nitrogen and stored at −80 °C until use. Separate samples of liver were snap-frozen using liquid nitrogen and stored at −80 °C for metabolomic analyses and Oil Red O histological staining. All animal studies were reviewed and approved by the Institutional Animal Care and Use Committee at the University of Nebraska (Lincoln, NE).

### Blood chemistry

After 2 weeks on the standard diet noted above, mice (control, C57BL/6, and *Fatp2*^−/−^) were fasted for 4 h (from 8 a.m. to 12 p.m.) and lightly anesthetized using isoflurane. Blood (100 μl) was collected by a retro-orbital bleed and immediately analyzed using Lipid Panel Plus Rotors in an Abaxis PICCOLO.

### Dietary fatty acid absorption

To address the role of FATP2 in the absorption of dietary fatty acids, 10-week-old control and *Fatp2*^−/−^ male mice (SV129 background, 12–14 mice/genotype) were fasted for 12 h followed by intraperitoneal injection with 500 mg/kg tyloxapol in PBS to inhibit lipoprotein lipase-dependent systemic fatty acid uptake (54). Mice were then given a bolus of flaxseed oil containing 500 mg/kg uniformly labeled [^13^C]oleate (C_18:1_) by gavage. Animals were sacrificed after 2 and 6 h, and blood was collected in EDTA-treated tubes. Total lipids were extracted from the whole blood using a modified Folch method with nonadecanoic acid (C_19:0_) (10 μg) as an internal standard and fatty acid methyl esters (FAMEs) prepared and resuspended in 100 μl of methyl acetate (see below) (55). FAMEs were analyzed using an Agilent 7890A GC system linked to an Agilent 5975C VL mass-selective detector (MSD) (Agilent, Palo Alto, CA) using electron impact ionization. GC was performed using an Agilent CP7421 Select FAME column, 200 m × 275 mm × 0.25 mm. Samples (1 μl) were injected in a splitless mode with selective ion monitoring. The MSD was set for selective ion monitoring of *m*/*z* 296 for endogenous ^12^C_18:1_ and *m*/*z* 314 for exogenous ^13^C_18:1_, using 100 ms of dwell time per ion.

### RNA isolation and quality controls

Liver slices (70 mg) snap-frozen in RNAlater® were thawed on ice, transferred to a 2-ml tube with 1 ml of QIAzol, and thoroughly homogenized. RNA was isolated using a standard QIAzol protocol (catalog no. 79306). After RNA isolation, we cleaned up the RNA using the Qiagen RNeasy kit following the manufacturer's protocol (catalog no. 74104). Quality control checks of the RNA were performed to define purity and concentration. A nanodrop (Nanodrop 1000, Thermo Scientific) spectrophotometer was used to assess the quantity and quality for each sample, ensuring that the quantity was between 50 and 250 ng/μl and the 260/280 was between 1.95 and 2.10. The lack of RNA degradation was confirmed using agarose gel electrophoresis for each sample. Prior to sequencing, Novogene performed additional quality and quantity assessments of the RNA samples using an Agilent 2100 instrument and gel electrophoresis.

### RNA-Seq and data analysis

RNA-Seq libraries were prepared using an Illumina TruSeq® RNA sample preparation kit as per the manufacturer's protocol. Library construction, additional quality assessment, and sequencing were performed by Novogene Corp., Ltd. Library concentration was quantified with a Qubit 2.0 fluorometer from Life Technologies, and samples were diluted to 1 ng/μl before checking insert size using a bioanalyzer (Agilent 2100 Bioanalyzer) and quantifying to greater accuracy using qPCR. Paired-end sequencing at a depth of 40 million reads or greater per sample was completed for data acquisition.

Data filtering was done to remove contaminants that contained adapters. Additionally, we discarded any reads that had greater than 10% uncertain nucleotides and greater than 50% low-quality nucleotides. Tophat2 was used to map the sequences to the C57BL/6 (*Mus musculus*) reference genome (GRCm38, mus_musculus_mm10). The mismatch parameter was set to 2, and all other parameters were set to default. HTSeq software was used to analyze the gene expression levels using union mode. We controlled for FDRs by using the Benjamini–Hochberg procedure based on negative binomial distribution in DESeq (25). Differentially expressed genes were obtained by performing pairwise comparisons between controls and *Fatp2*^−/−^ mice for both sexes separately. For pathway enrichment, we selected for genes that maintained an adjusted *p* value of ≤0.05. KEGG pathway enrichment analysis used the David Bioinformatics Database and resources. The enriched KEGG pathways focused on for this study were selected by their completeness of enrichment and a corrected *p* value of ≤0.05.

### Histology

Oil Red O staining was performed by the University of Nebraska Medicine Tissue Science Facility. The flash-frozen tissue was sectioned at 5–10 μm thick and mounted on slides. Slides were air-dried for 30–60 min at room temperature and then fixed in ice-cold 10% formalin for 5–10 min. After formalin fixation, the slides were rinsed immediately in three changes of distilled water and then air-dried again for 30–60 min. Dried slides were placed in absolute propylene glycol for 2–5 min to avoid carrying water into Oil Red O. Slides were then stained in prewarmed Oil Red O solution for 8–10 min in a 60 °C oven. The stained slides were differentiated in 85% propylene glycol solution for 2–5 min and then rinsed in two changes of distilled water. After rinsing, the slides were stained in Gill's or Mayer's hematoxylin for 30 s and then washed in distilled water for 3 min. The slides were placed in distilled water and then mounted with glycerin jelly medium. Image acquisition was done with a Nikon Ti-2 inverted fluorescence microscope. Lipids stained in red were quantified using ImageJ version 1.5 on Windows 10. The total area of red was quantified using the following thresholds in ImageJ: hue, 195 and 255; saturation, 0 and 255; brightness, 0 and 255; threshold method, default; threshold color, red; color space, HSB). The area of red pixels was quantified using three images for each liver sample, and the statistical analysis was done in Microsoft Excel using Student's *t* test.

### Quantitative RT-PCR

For cDNA preparation, RNA was reverse-transcribed into cDNA using the iScript cDNA synthesis kit as detailed by the manufacturer (Bio-Rad). The PCR conditions were set according to the manufacturer's instructions. Bullseye EvaGreen qPCR 2× Mastermix from MidSci was used in a Mastercycler EP Realplex (Eppendorf). All primers were custom-synthesized and purchased from Integrated DNA Technologies, Inc. All primers used are listed in Table 4. *Gapdh* was used as the housekeeping gene for data normalization. We used normalized expression values from RT-qPCR experiments and calculated log_2_ FC of 15 genes (30 measurements total) by dividing *Fatp2*^−/−^ expression values by control expression and converting to a logarithmic scale (log_2_ FC = log_2_(*Fatp2*^−/−^ normalized value/control normalized value)). These were plotted against the log_2_ FC values of the corresponding genes from RNA-seq data.

**Table 4:** **Primers used for RT-qPCR**

Gene	Forward primer	Reverse primer
*Gapdh* (NM_008084.2)	F1: 5′-TCA ACA GCA ACT CCC ACT CTT CCA-3′	R1: 5′-ACC ACC CTG TTG CTG TAC CGT ATT-3′
*Fatp1* (NM_011977.3)	F1: 5′-TGG TCA AGG TCA ATG AGG ACA CGA-3′	R1: 5′-ACG CTG TGG GCA ATC TTC TTG TTG-3′
*Fatp2* (NM_011978.2)	F1: 5′-ACA CAC CGC AGA AAC CAA ATG ACC-3′	R1: 5′-TGC CTT CAG TGG ATG CGT AGA ACT-3′
*Fatp3* (NM_011988.2)	F1: 5′-AGC TGC TGA AGG ATG TCT TCT-3′	R1: 5′-TCC AAG ACC TCA GCC ACT TCA GTT-3′
*Fatp4* (NM_011989.4)	F1: 5′-AGT AAG CAT GTG GCT TTG GGC AAG-3′	R1: 5′-TTT GGC AGA AGA TGG AGC AAC AGC-3′
*Fatp5* (NM_009512.2)	F1: 5′-TGT AAC GTC CCT GAG CAA CCA GAA-3′	R1: 5′-ATT CCC AGA TCC GAA TGG GAC CAA-3′
*Fatp6* (NM_001081072.1)	F1: 5′-TTG GGA CCG TCT TGG AGA CAC TTT-3′	R1: 5′-TGC TTC CTG GAT GAA GTC CAA CCT-3′
*Acsl1* (NM_007981.3)	F1: 5′-TGC AGC GAG TGT GGG AAA G-3′	R1: 5′-TGG TAA GAC CCC GTG GAC-3′
*Acsl5* (NM_027976.2)	F1: 5′-TCG ATG CAA TGC CTG CAC T-3′	R1: 5′-TGC AGG GAC TGA AGG CCA-3′
*Acsl6* (NM_001033597.1)	F1: 5′-AGT CTC CGT GTG AAG CTC CAG-3′	R1: 5′-TCA CGA ATA ACT ATA ATA TTG CTG CAA A-3′
*SERPINb6* (NM_001164117.1)	F1: 5′-ACG GTG AGG TGC ATG AGA TTC ACT-3′	R1: 5′-AGC AAA CCA TGG AGA GAG GGT CAA-3′
*Fabp1* (NM_017399.4)	F1: 5′-AGT CGT CAA GCT GGA AGG TGA CAA-3′	R1: 5′-GAC AAT GTC GCC CAA TGT CAT GGT-3′
*Fabp2* (NM_007980.2)	F1: 5′-AAA GGA GCT GAT TGC TGT CCG AGA-3′	R1: 5′-TCG CTT GGC CTC AAC TCC TTC ATA-3′
*Npc1L1* (NM_207242.2)	F1: 5′-CAG CAC TGG CCA CTT TGT TGT CAT-3′	R1: 5′-TGT TCC ACA CCC TAT TTC CTG CCT-3′
*Fasn* (NM_007988.3)	F1: 5′-TAT CCT GCT GTC CAA CCT CAG CAA-3′	R1: 5′-TCA CGA GGT CAT GCT TTA GCA CCT-3′
*Cyp4A10* (NM_010011.3)	F1: 5′-CAGGAAATTGTGTCGTGCATAG-3′	R1: 5′-ACTTTCATGTAGTCAGGGTCATAG-3′
*Cd36* (NM_001159555.1)	F1: 5′-ACT GGG AAA ATC AAG CTC CTT G-3′	R1: 5′-TGA AAT CAT AAA AGC AAC AAA CAT CA-3′

### Total fatty acid analysis

Total lipids were isolated from liver tissue using a modification of the Folch method (55). Liver tissue (50 ± 1.0 mg) was thawed on ice and homogenized in 3 ml of chloroform/methanol (2:1, v/v) containing 0.05% butylated hydroxytoluene. Afterward, 100 μg of C_19:0_ was added as an internal standard. Samples were shaken at 25 °C for 1 h and clarified by centrifugation (2,500 × *g*, 10 min). The supernatant was transferred a new tube, 0.25 volumes of water were added, and samples were vortexed for 10 s and clarified by a second round of centrifugation. The lower phase was collected and dried down under a stream of nitrogen. 0.5 ml of 1% sulfuric acid in methanol and 0.25 ml of toluene was added, and the samples, thoroughly mixed, were sealed under nitrogen incubated for 12 h at 50 °C in a heating block. Samples were cooled to room temperature, 1.25 ml of 5% NaCl was added, and the samples were thoroughly mixed. The FAMEs were extracted from the samples twice using hexane. The upper hexane layer was collected and washed with 1 ml of 2% potassium bicarbonate, and FAMEs were collected as the flow-through from a column containing anhydrous sodium sulfate. The samples were dried under nitrogen and resuspended in 100 μl of methyl acetate, and total fatty acid profiles (as FAMEs) were analyzed with an Agilent 7890A GC system linked to an Agilent 5975C VL MSD, using electron impact ionization equipped with an Agilent CP7421 Select FAME column (200 m × 275 mm × 0.25 mm).

### Neutral lipid quantification

Total lipids were isolated from liver tissue using a modification of the Folch method (55). Liver tissue (∼50 mg) was thawed on ice and homogenized in 3 ml of chloroform/methanol (2:1, v/v) containing 0.05% butylated hydroxytoluene. 100 μg of triglyceride standard C_17:0_ was added as an internal standard. Samples were shaken at 25 °C for 1 h and clarified by centrifugation (2,500 × *g*, 10 min). The supernatant was transferred a new tube, 0.25 volumes of water were added, and samples were vortexed for 10 s and clarified by a second round of centrifugation. The lower phase was collected and dried down under a stream of nitrogen. 1.0 ml of methylene chloride was used to resuspend the extract. To select for neutral lipids, the extract was passed through columns made using a glass pipette packed with glass wool, high-purity-grade silica gel (60 Å, 200–425-mesh particle size, CAS: 112926-00-8), and 300 mg of 0.1-mm-diameter zirconia/silica beads. After the extract was passed through the column, it was dried using a nitrogen evaporator, resuspended again in 0.5 m potassium hydroxide, and incubated for 5 min at 100 °C. After incubation, 12% boron trifluoride was added and incubated for 5 min at 100 °C. 0.5 ml of hexane and 2 ml of saturated NaCl in water was added. Samples were centrifuged at 10,000 rpm for 10 min, and the top layer was collected for analysis in GC/MS. Total triglyceride profiles were quantified from the FAME profiles generated using an Agilent 7890A GC system linked to an Agilent 5975C VL MSD (mass-selective detector), using electron impact ionization equipped with an Agilent CP7421 Select FAME column (200 m × 275 mm × 0.25 mm).

### Targeted metabolomics to define bioactive lipids

Liver tissue samples were suspended in 500 μl of 80% acetonitrile in water (20%) by volume. As an internal control, 200 μg of 20-HETE-*d*_6_ (Cayman Chemicals) was added to each sample. The samples were kept on ice for 1 h and disrupted using a Bullet Blender (Next Advance, 3 cycles of 3 min at a power setting of 7) using 0.5-mm ZrO beads. The samples were clarified by centrifugation (15,000 × *g*, 10 min), and the supernatants were transferred to new tubes, dried, and resuspended into 50 μl of 80% acetonitrile in HPLC grade water. For metabolite quantification, samples (5 μl) were separated using an Amide XBridge column (4.6 × 100 mm, Waters Millipore, Waltham, MA) at a flow rate of 0.5 ml/min (stationary phase (*n*-propylamine); first mobile phase (100% acetonitrile); second mobile phase (20 mm NH4OAc, pH 9.50)) linked to a 4000QTrap (Sciex, Framingham, MA) operating in the negative MRM mode. A mixture of eicosanoid standards was used for identification and to determine elution times: (+)5-HETE, (+)8-HETE, (+)11-HETE, (+)12-HETE, and hexadeuterated (*d*_6_) 20-HETE (Cayman Chemicals) dissolved into 80% methanol over a range of concentrations (1–10 μg/ml). The relative concentrations of the different eicosanoids identified were normalized to the internal 20-HETE-*d*_6_ standard; protein concentrations were determined using the Pierce^TM^ BCA protein assay kit per the manufacturer's instructions (Bio-Rad).

### Statistical analyses

A minimum of four animals were used for each analysis. Statistical significance was determined using a two-tailed paired *t* test. Significance was selected for using a maximum *p* value of ≤0.05. To determine statistical significance that controls for false discovery rates in differentially expressed genes, we performed a Benjamini–Hochberg method based on negative binomial distribution in DESeq. Statistical significance was selected for by using an adjusted *p* value of ≤0.05.

## Data availability

All of the data are contained within the article with the exception of the primary RNA-Seq data sets, deposited in the NCBI Gene Expression Omnibus (54) and accessible through GEO Series accession number GSE140147. All data will be shared upon request to Paul N. Black (pblack2@unl.edu).

## Author contributions

V. M. P., C. C. D., and P. N. B. conceptualization; V. M. P. and N. W. data curation; V. M. P., M. B., N. W., and C. C. D. formal analysis; V. M. P., C. C. D., and P. N. B. funding acquisition; V. M. P., J. G., and N. W. investigation; V. M. P., J. G., M. B., N. W., C. C. D., and P. N. B. methodology; V. M. P. and P. N. B. writing-original draft; V. M. P., J. G., M. B., N. W., C. C. D., and P. N. B. writing-review and editing; M. B. validation; C. C. D. and P. N. B. supervision; C. C. D. visualization; C. C. D. and P. N. B. project administration.

## Supplementary Material

Supporting Information

## Footnotes

2 The abbreviations used are:

FATP2: fatty acid transport protein 2
PPAR: peroxisome proliferator–activated receptor
FA: fatty acid
PMN-MDSC: polymorphonuclear myeloid-derived suppressor cell
DEG: differentially expressed gene
PCA: principal component analysis
FDR: false discovery rate
TIFA: TNFα receptor–associated factor (TRAF)-interacting protein with a forkhead-associated (FHA) domain
qPCR: quantitative PCR
PGJ2: prostaglandin J2
LXA4: lipoxin A4
VLCFA: very-long-chain fatty acid
FAME: fatty acid methyl ester
HETE: hydroxyeicosatetraenoic acid.
