# Supplementary material for: Deletion of fatty acid transport protein 2 (FATP2) in the mouse liver changes the metabolic landscape by increasing the expression of PPARα-regulated genes
Source: J Biol Chem. 2020 Mar 18;295(17):5737–50. doi: 10.1074/jbc.RA120.012730 (PMC7186177; doi:10.1074/jbc.RA120.012730)
Supplement: Supporting Information [file supp_295_17_5737__index.html]

Deletion of Fatty Acid Transport Protein 2 (FATP2) in the mouse liver changes the metabolic landscape by increasing the expression of PPARα-regulated genes — FATP2 expression is linked to PPARα-regulated genes in liver — Deletion of fatty acid transport protein 2 (FATP2) in the mouse liver changes the metabolic landscape by increasing the expression of PPARα-regulated genes — FATP2 expression is linked to PPARα-regulated genes in liver — Supporting Information 

# Deletion of fatty acid transport protein 2 (FATP2) in the mouse liver changes the metabolic landscape by increasing the expression of PPARα-regulated genes

## Supporting Information

- Table S1 - Read counts and FPKM values of the individual Fatp2-/- mice.
- Table S2 - Read counts and FPKM values of the individual control mice.
- Table S3 - Differential expression analysis of the Fatp2-/- male and female mice.
- Table S4 - The identified differentially expressed genes from the male and female Fatp2-/- mice.
- Supplemental Tables and Figure - The Supplemental Tables are from the RNA-Seq experiments and are uploaded as 4 separate Excel spreadsheets and so noted
