# Supplementary material for: Deletion of fatty acid transport protein 2 (FATP2) in the mouse liver changes the metabolic landscape by increasing the expression of PPARα-regulated genes
Source: J Biol Chem. 2020 Mar 18;295(17):5737–50. doi: 10.1074/jbc.RA120.012730 (PMC7186177; doi:10.1074/jbc.RA120.012730)
Supplement: Supporting Information [file supp_RA120.012730_158343_2_supp_491561_q716nk.pdf]

Supplemental Tables and Figures

Table S1. (Excel File, *attached*) **Read counts and FPKM values of the individual *Fatp2-/-* mice.** The experimental setup is on the excel sheet 1. The read counts of the *Fatp2-/-* mice before differential expression analysis are on excel sheet 2, and the fragments per kilobase of transcript per million mapped reads (FPKM) are on excel sheet 3. *Fatp2-/-* mice include the males (629, 638, 650, and 693) and the females (631, 643, 634, and 652).

Table S2. (Excel File, *attached*) **Read counts and FPKM values of the individual control mice.** The experimental setup is on the excel sheet 1. The read counts of the control mice before differential expression analysis are on excel sheet 2, and the fragments per kilobase of transcript per million mapped reads (FPKM) are on excel sheet 3. Control mice include the males (695, 662, 675, and 655B) and the females (656, 659, 676, and 684).

Table S3. (Excel File, *attached*) **Differential expression analysis of the *Fatp2-/-* male and female mice.** The experimental setup is on the excel sheet 1. Differential expression analysis is on sheet 2 and contains the gene name id, gene name, chromosomal location of the gene, strand sense, start and stop location of the gene, length of the gene, and gene description (columns A – H). Average readcount of the *Fatp2-/-* and control male mice are in columns I and J. The $\log_2$ fold-change, p-value, adjusted p-value, and significance (TRUE = $p \leq 0.05$, FALSE = $p \geq 0.05$) of the *Fatp2-/-* male mice is in columns I - N. Female *Fatp2-/-* data is in the same order as the males in columns O – T.

Table S4. (Excel File, *attached*) **The identified differentially expressed genes from the male and female *Fatp2-/-* mice.** The differentially expressed genes are in their corresponding sheets. For example, the sheet labeled "(Upregulated DEGs Male)" contains the genes in the *Fatp2-/-* mice that had an increased fold-change and adjusted p-value≤0.05. DEGs were selected using an adjusted p-value cut-off of 0.05.

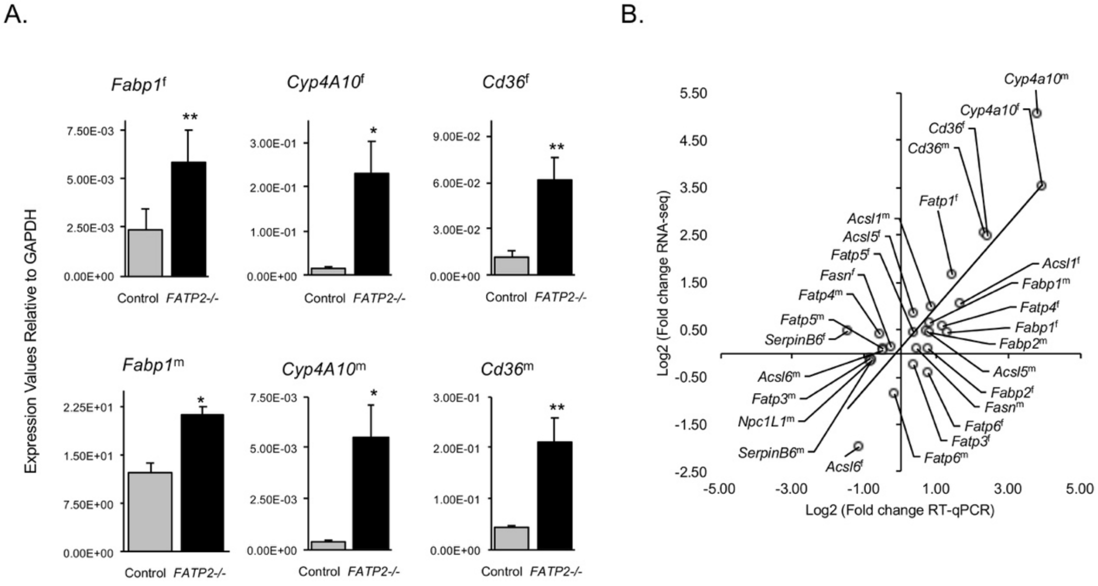

Figure S1. Changes in gene expression in the *Fatp2*$^{-/-}$ liver using RT-qPCR. A. RT-qPCR data shown is from female (upper) and male (lower) liver tissue. Transcripts for FABP1, CYP4A10, and CD36 were found in higher abundance in the *Fatp2*-/- liver tissue of both female and male mice ± SD (N=4), **p*≤0.05, ***p*≤0.01 from a student's t-test. Superscripts m and f correspond to male and female tissue, respectively. B. Using RT-qPCR and RNA-sequencing data, correlation of the Log_2 FC values of 15 genes in male and female tissues was determined (30 measurements total). The expression values from the two sources of data correlated to R^2=0.74 from the linear trendline. Superscripts m and f correspond to male and female liver, respectively.
